# Soft Robotic Deployable Origami Actuators for Neurosurgical Brain Retraction

**DOI:** 10.3389/frobt.2021.731010

**Published:** 2022-01-14

**Authors:** Tomas Amadeo, Daniel Van Lewen, Taylor Janke, Tommaso Ranzani, Anand Devaiah, Urvashi Upadhyay, Sheila Russo

**Affiliations:** ^1^ Mechanical Engineering Department, Boston University, Boston, MA, United States; ^2^ School of Medicine, Boston University, Boston, MA, United States

**Keywords:** surgical robotics, soft robotics, origami robots, neurosurgery, retraction

## Abstract

Metallic tools such as graspers, forceps, spatulas, and clamps have been used in proximity to delicate neurological tissue and the risk of damage to this tissue is a primary concern for neurosurgeons. Novel soft robotic technologies have the opportunity to shift the design paradigm for these tools towards safer and more compliant, minimally invasive methods. Here, we present a pneumatically actuated, origami-inspired deployable brain retractor aimed at atraumatic surgical workspace generation inside the cranial cavity. We discuss clinical requirements, design, fabrication, analytical modeling, experimental characterization, and *in-vitro* validation of the proposed device on a brain model.

## 1 Introduction

New cases of brain tumors in 2021 are estimated to be up to 24,530 leading to 18,600 deaths in the US alone (Siegel et al., 2021). Neurological surgery is a highly specialized field: surgery for diseases and disorders in these areas include tumor resection, vascular derangements, functional surgery, and other indications. Surgical treatment in these anatomic locations requires navigating and manipulating very sensitive structures that are often difficult to access and/or intolerant to manipulation (Ganly et al., 2005). Significant developments in minimally invasive brain surgery have been progressively moving surgical techniques towards minimizing tissue trauma while expanding the scope of surgical options to enable better results, shorter recovery time, and better quality of life in the short and long term (Nicolai et al., 2008; Devaiah and Andreoli, 2009; Eloy et al., 2009; Fu et al., 2016; Rawal et al., 2016).

Robotic surgery approaches have been employed in neurosurgical procedures and have demonstrated potential utility (Iwata et al., 2011; Bertelsen et al., 2013; Hannaford et al., 2013; Smith et al., 2016). A preclinical cadaver study was conducted to assess the feasibility of performing robotic-assisted neurosurgery with the da Vinci Surgical System (Intuitive Surgical, Inc.); however, design constraints such as size and rigidity of the robotic instrumentation presented limitations (Blanco and Boahene, 2013). The Robotic Stereotactic Assistance Device (ROSA, Medtech S.A.), has been employed in pediatric neurosurgery for epilepsy treatment, depth electrode placement, and ventriculostomy (Hoshide et al., 2017; Nelson et al., 2020). Currently, few robotic platforms designed for neurosurgical and skull base surgery applications are commercially in use and many other robotic systems for neurosurgery are in the research and development stage (Smith et al., 2016). A meso-scale dexterous robot for intracranial neurosurgery (MINIR), with multiple rigid links actuated by shape memory alloy wires and constructed out of MRI compatible materials, was proposed in (Ho et al., 2012). This device was experimentally validated *in-vitro* using a gelatin medium. A recent development of the MINIR system was focused at creating a continuum-like robot body and providing a more compliant interface with brain tissue (Kim et al., 2017). Other research groups are focusing on robotic platforms for minimally invasive access to deep-seated brain lesions. Such approaches consist in inserting instruments though the nostrils and navigating inside the brain, and are employed in specific applications of neurosurgery and skull base surgery (e.g., pituitary surgery, cranial base tumors, intraparenchymal lesions). A telerobotic system based on concentric tube continuum robots was developed for pituitary surgery and validated in cadaver tests (Burgner et al., 2014). The concentric tube robotic architecture was also proposed by the same research group to remove intracerebral hemorrhages in a minimally invasive fashion (Swaney et al., 2013). All of the aforementioned robotic platforms for neurosurgery have shown capabilities in direct tissue manipulation, navigation, and therapy delivery; however, retraction of brain tissue is not enabled by these systems.

Surgical retraction, i.e. displacement of neural and neurovascular structures to access deeper locations in the cranium or to create workspace for other surgical tools, is performed using straight rigid plastic or metal tools (i.e., retractors) (Assina et al., 2014). These instruments can generate excessive pressure on the anatomical structures during surgery and consequent tissue damage, thus leading to neurological and other functional impairments post-surgery (Zhong et al., 2003). This is mostly caused by the inadequacy of traditional neurosurgical tools to match in compliance with surrounding biological tissues (Mansour et al., 2020).

Brain retraction systems are frequently required to achieve surgical exposure of deep-seated brain lesions (Marenco-Hillembrand et al., 2020). Retraction, however, can cause localized stress areas and is associated with complications that include brain edema, vascular compromise causing ischemia, and direct damage to the surrounding cortex (Zhong et al., 2003). The frequency of retraction-induced injury has been estimated to be approximately 10% in skull base surgery and 5% in intracranial aneurysm operations (Andrews and Bringas, 1993). Modern retractor designs have improved ease of use and minimized, but not yet entirely eliminated, retraction-induced injuries (Zagzoog and Reddy, 2020). Types of retraction include spatula-based and tubular retractors (Assina et al., 2014; Eichberg et al., 2020; Shapiro et al., 2020). Spatula-based systems can be associated with injury to the cortex and deep white matter, particularly adjacent to the sharp edges, which can result in uneven pressure on the parenchyma over the course of long operations. Tubular retractors may reduce damage to surrounding tissues; however, they still cause cytotoxic edema and cellular damage (Bander et al., 2016). Ad-hoc robotic solutions to solve clinical issues caused by brain tissue retraction have not been developed yet.

Soft robotic approaches are recently making their entrance in the field of robotic-assisted neurosurgery. A ferromagnetic soft continuum robot has been proposed for minimally invasive robotic surgery in hard-to-reach areas, such as the cerebrovascular structures. (Kim et al., 2019). The robot is composed of uniformly dispersed ferromagnetic microparticles in a soft polymer matrix. Its navigation capabilities were assessed in an *in-vitro* phantom model.

Soft robots have great potential in neurosurgical applications, particularly for brain tissue manipulation and retraction tasks. They present the opportunity to interact more gently and safely manipulate biological living tissues and preserve their physiological functions (Runciman et al., 2019; Roche et al., 2017).

**FIGURE 1:**
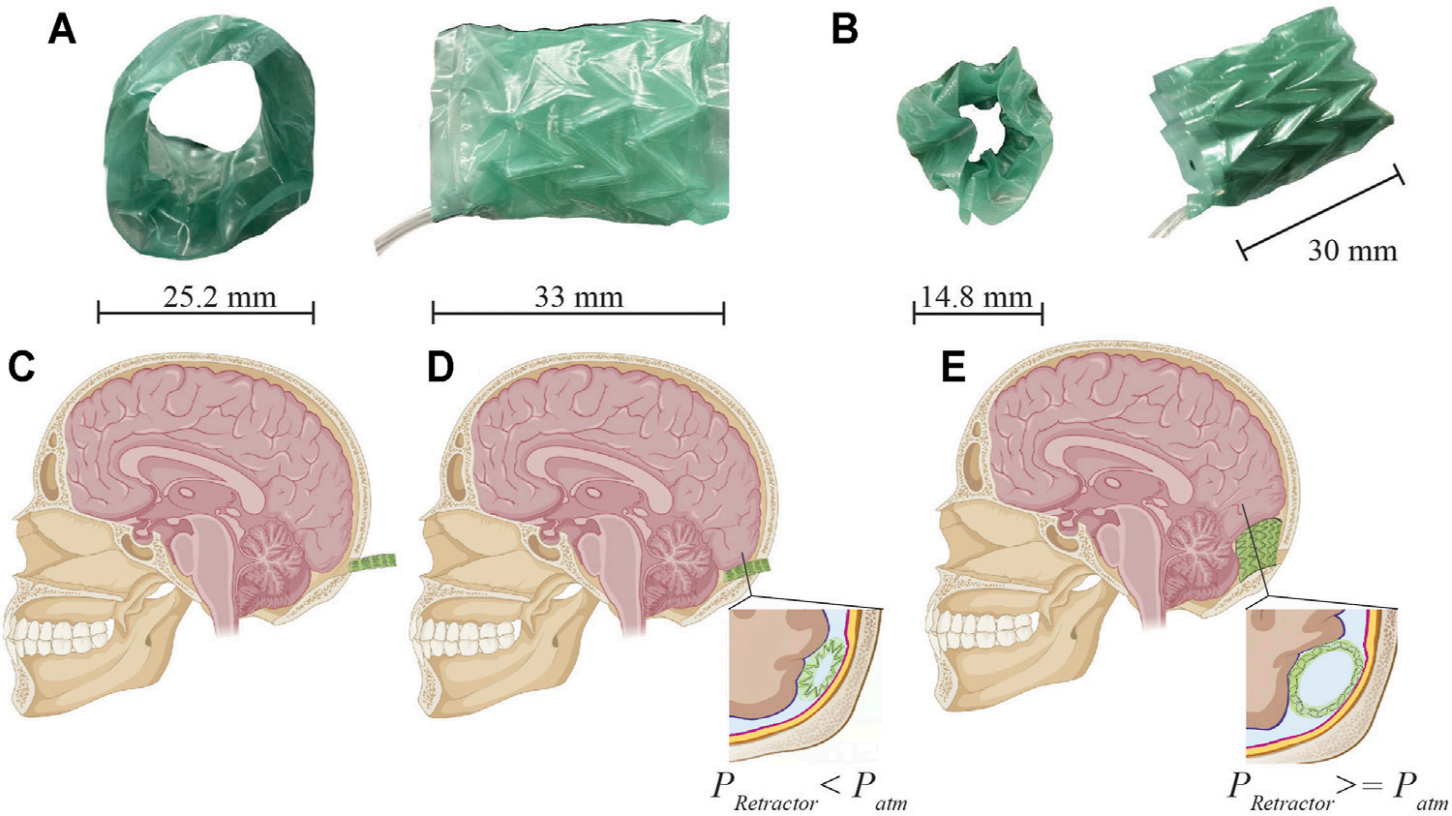
Soft robotic retractor prototype and conceptual schematic of clinical application in neurosurgery. **(A–B)** Prototype front and side views in expanded **(A)** and contracted **(B)** configurations. **(C–D)** Neurosurgical application. **(C)** A bore hole drilled into the cranial cavity allows the device to be inserted in its contracted configuration (minimum diameter). **(D)** Placement of the device against the rigid surface of the cranium. **(E)** Expansion of the device displaces brain tissue which opens a passage for other surgical tools and access to the surgical site.

In this paper, we present a first proof-of-concept of a soft robotic retractor (Figure 1). The proposed system is designed to facilitate neurosurgical operations by creating workspace in the cranial cavity through expansion of pneumatically driven origami deployed actuators. We discuss clinical requirements, design, fabrication, analytical modeling, experimental characterization, and *in-vitro* validation of the proposed device on a brain model. In contrast to traditional passive retractors, our system provides the capability to tune the amount of retraction via actively expanding the structure by controlling its internal pressure. Similar expansion function has been seen in medicine through the use of stents for a broad range of endovascular procedures throughout the body (to increase the lumenal diameter of vasculature). However, unlike a stent which is deployed and irreversibly expanded, the proposed robotic device can actively control its expansion and contraction, and is not intended for long term implantation.

## 2 Design and Fabrication

### 2.1 Clinical Requirements

The proposed soft robotic retractor needs to operate safely and effectively in the cranial cavity. A number of functional requirements are associated with this application including tissue displacement, force output, and biocompatibility.

The retractor must be designed to collapse to a small footprint for insertion into the cranium. As a clinically relevant benchmark for minimum diameter, a drilled bore hole would meet this requirement. Hence, the retractor should collapse to less than 15 mm in diameter. Expansion of the device should create appreciable workspace similar to current procedures. A review of surgical brain retraction shows this can range from 12 to 28 mm (Shapiro et al., 2020), with excised tumors ranging from 15 to 29 mm in diameter. The retractor should fall within this range of surgical brain retraction values to open up a passage for accommodating neurosurgical instrument operation and excision of tumors.

Force output of the device is another important clinical requirement for expansion and displacement of tissue. Intraoperative forces during transnasal endoscopic tumor excision in an *in-vivo* experiment ranged from 0.1 to 0.5 N, with peak maximal forces up to 2.12 N (Bekeny et al., 2013). Other studies of tool and brain tissue interactions showed a range of forces between 0.5–6 N (Aggravi et al., 2016). A review of 13 studies found mean maximum force output from hand tools in neurosurgery to be 1.48 N. Similarly, the review found that three studies reported a mean maximum force output from retraction tasks to be 2.5 N (Golahmadi et al., 2021). The soft retractor should target force output values in a similar range with a maximum of 3 N to generate forces comparable to current retraction procedures and ensure safety.

Materials for the construction of the soft robotic retractor and actuation methodologies should guarantee safety and biocompatibility of the device in surgical operations.

A soft biocompatible elastomer should enshroud the device for safe contact with brain tissue. Soft fluidic actuation should be used in the retractor to provide a safeguard for ruptures or failure of the device. In such a case, the retractor may simply leak a safe, saline solution or air into the surgical site. This kind of soft fluidic actuation (i.e., hydraulic or pneumatic) has been widely adopted in soft robots designed for surgical applications (Runciman et al., 2019; Cianchetti et al., 2018; Gifari et al., 2019). We selected pneumatic actuation via air gas for the soft robot. The neurosurgical procedures targeted by the robotic retractor operate via an open cavity in the cranium which is exposed to air at atmospheric pressure allowing for a vent in the case of a leak.

### 2.2 Design

**FIGURE 2:**
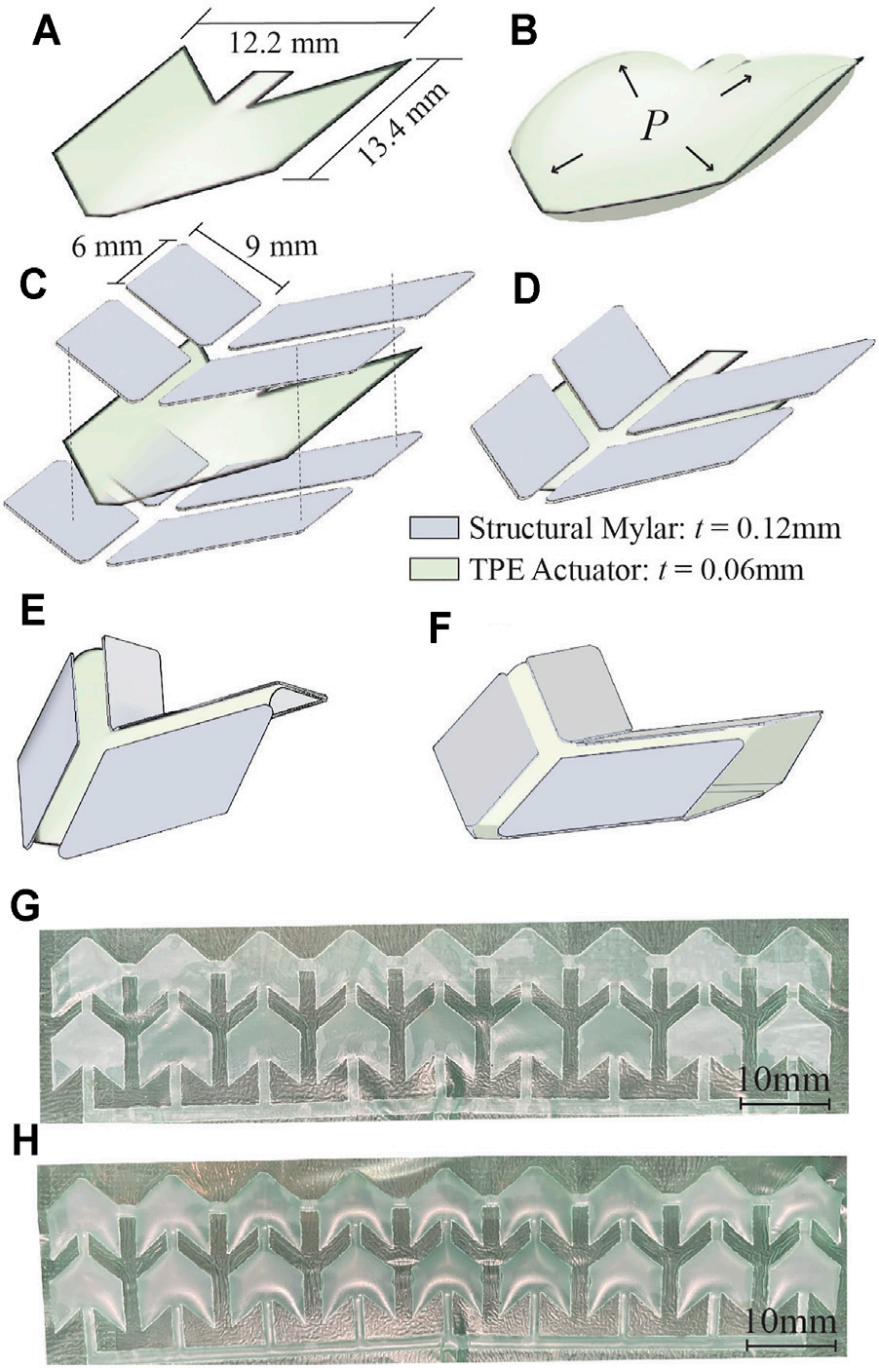
Design of integrated pneumatic pockets into the origami structure. **(A)** Individual inflating pockets are heat and pressure-sealed in the shape of a single Miura-Ori pattern. **(B)** Positive pressure delivered to the TPE actuator causes these pockets to inflate. **(C–D)** Assembly of structural Miura-Ori pattern over the inflatable pocket. **(E)** Folded origami with integrated inflating pocket. **(F)** Application of positive pressure to the pocket causes unfolding of the structural origami pattern. **(G–H)** 18 unit array of pneumatic pockets (shown here not integrated with origami structure) at atmospheric pressure **(G)** and pressurized at 40 kPa **(H)**.

The soft robotic retractor relies upon pneumatic driven actuation of an origami structure. Expandable soft origami robotic structures have been proposed in (Chauhan et al., 2021; Becker et al., 2017; Li et al., 2017; Ranzani et al., 2017; Martinez et al., 2012; Russo et al., 2017; Rus and Tolley, 2018; Paez et al., 2016; Russo et al., 2016). The origami-inspired folding design creates a deployable device, capable of expansion in the brain cavity. Both positive and vacuum pressures are delivered to pneumatic chambers integrated into the device to provide actuation. We deliver vacuum pressures to an origami skeleton which has been sealed inside of a thermoplastic elastomer (TPE) bag. The tightly wrapped TPE film forms actuation chambers in the valley regions of the origami structure (Figures 1A,B). When a vacuum is applied, the differential pressure between the chamber and external atmospheric pressure drives the elastomer film to cling to the origami structure. The resultant force pulls the origami skeleton into a contracted configuration. In this contracted configuration, the retractor has an outer diameter of 14.8 mm (Figure 1B). The surgeon will position the retractor using standard surgical instrumentation (Figure 1C). The retractor will then expand, anchoring itself onto the cranium bones, and displace brain tissue to create a passage for other tools to access the surgical site of interest (Figures 1D,E). Expansion of the retractor is achieved by releasing vacuum pressure and letting the device expand to atmospheric pressure. Building on this method, we integrate inflatable pneumatic chambers into the origami structure to achieve further unfolding (Figure 2).

The soft robotic retractor is designed as a multi-layer assembly of thin biocompatible polymeric films. It consists of an origami-based central structure (with Miura folds), that can fold and unfold upon application of negative and positive pressure, respectively. Each origami unit of the retractor is equipped with inflatable pockets (i.e., pneumatic actuators) that can inflate between creases and further unfold the structure (Figure 2F). The origami structure is subdivided into 18 unit cells (Figure 2G). Each structural element measures 9×6 mm (Figure 2C). Four structural elements make up a cell for a total of 72 elements. A 0.8 mm wide gap is included between all elements. The inflatable pockets are placed within each cell and measure 12.2×13.4 mm, shown in Figure 2A. With the application of pressure we can drive the origami sheet from a folded configuration (Figure 2E) to an unfolded one (Figure 2F). A single rectangular sheet of this origami measures 90×31 mm.

The assembled cylindrical retractor measures 25.2 mm in outer diameter when fully expanded and 14.8 mm when fully contracted with vacuum pressure (Figures 1A,B). The total length of the device may be adjusted during fabrication to better accommodate specific patient anatomy or for specific surgical procedures, based on feedback from the surgeons. The retractor is constructed from layers of thin plastic films with a total thickness of 0.52 mm. Expansion of the device opens a passage for surgical tools similarly to traditional non-robotic retractors. A review of surgical brain retraction shows this can range from 12 to 28 mm (Shapiro et al., 2020). The retractor falls within this range of surgical brain retraction values to open up surgical workspace for accommodating neurosurgical instrument operation and excision of tumors.

### 2.3 Fabrication

**FIGURE 3:**
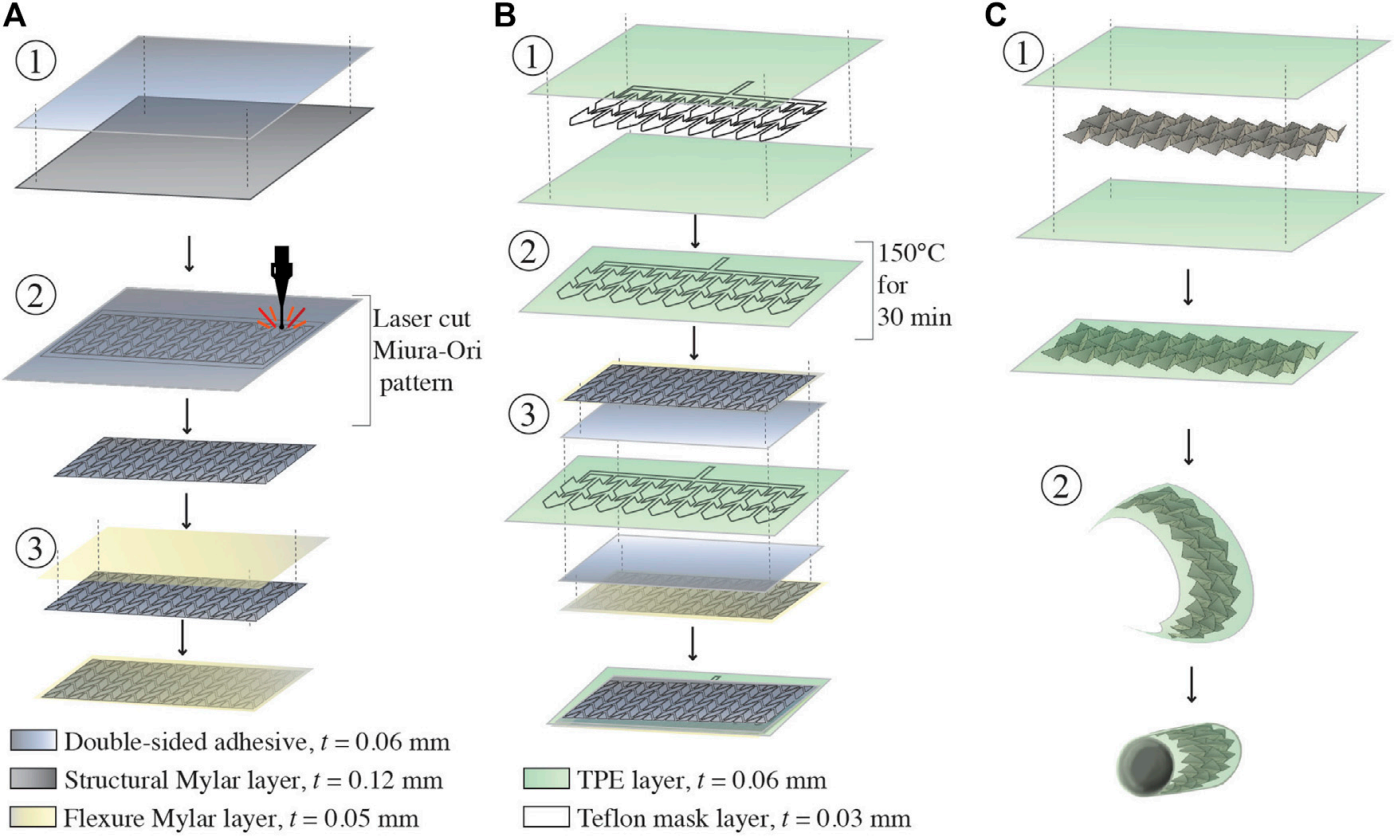
Fabrication of the soft robotic retractor. **(A)** Assembly of the Miura-Ori origami structure. Double sided adhesive film is placed on a layer of structural Mylar and laser cut. **(A)** Flexural layer of Mylar is applied onto the exposed adhesive surface of the origami patterned assembly. **(B)** Assembly of the pneumatic actuators. Two layers of TPE are heat and pressure bonded in desired locations using a laser cut Teflon mask. The Mylar origami structure and TPE pneumatic actuators are assembled together with a layer of double-sided adhesive. This is done on both sides of the TPE actuators. **(C)** Final assembly of the retractor. The assembly is manually folded into the Miura-Ori pattern. The assembly is sealed in a tightly wrapped TPE vacuum actuation bag. The assembled retractor sheet is wrapped into a cylinder and flexible adhesive is used to secure the cylinder along one seam.

Construction of the retractor involves the assembly of two different layers of Mylar (Grafix Platics, Maple Heights OH, United States). The flexural layer is 0.05 mm thick. The origami elements are comprised of structural Mylar, 0.12 mm thick, adhered to the flexural layer using 0.06 mm thick adhesive film (467 MP, 3 M, St.Paul MN,United States). First, the structural Mylar layer is prepared with the double sided adhesive (Figure 3A, step 1). The origami pattern is cut into the Mylar sheet using a 40 W *C O*_2_ laser cutter (Glowforge, Seattle WA, United States) which creates 72 individual structural elements (Figure 3A, step 2). Afterwards, the flexural layer is adhered to the origami structural elements (Figure 3A, step 3). The flexural layer binds together the structural elements and allows creasing at specific locations which enables the origami pattern to develop.

The pneumatic actuators component is constructed from layers of 0.03 mm thick TPE (Stretchlon 200, FibreGlast, Brookville OH, United States) and 0.03 mm thick Teflon. The Teflon layer is laser cut to the desired shape (Figures 2G,H) of the actuators and sandwiched between two layers of TPE (Figure 3B, step 1). Then, the TPE layers are sealed together by placing the assembled layers of TPE and Teflon into a clamped fixture and heated in an oven at 150°C for 30 min (Figure 3B, step 2). The Teflon serves as a mask providing a layer of insulation, preventing the TPE layers from bonding in selected areas as they are heat-pressure bonded, similarly to (Becker et al., 2017; Ranzani et al., 2017). Pneumatic inlet tubes of 2 mm in diameter (Grayline Inc. Waukesha WI, United States) are sealed with flexible adhesive (Loctite 13,60 ,694, Hartford CT, United States) to inflate the pneumatic actuators.

The retractor is assembled by adhering two sheets of Miura-Ori origami onto either side of the TPE pneumatic actuators, as shown in Figure 3B, step 3. Finally, the structure is folded into its origami configuration and sealed inside of a TPE bag (Figure 3C, step 1). This bag forms the sealed chamber for vacuum contraction actuation of the retractor. The retractor is folded into a hollow cylinder (thickness of 0.52 mm) and a single seam is adhered with flexible adhesive (Loctite 13,60 ,694, Hartford CT, United States), as shown in Figure 3C, step 2. Another pneumatic inlet tube of 3 mm in diameter (Grayline Inc. Waukesha WI, United States) is sealed to the assembly to provide vacuum pressure.

### 2.4 Modeling

As described in Section 2.2; Section 2.3, the origami structure of the soft foldable robotic retractor is constructed of rigid and flexible layers so that it can be folded at specific locations where flexure joints are created.

**FIGURE 4:**
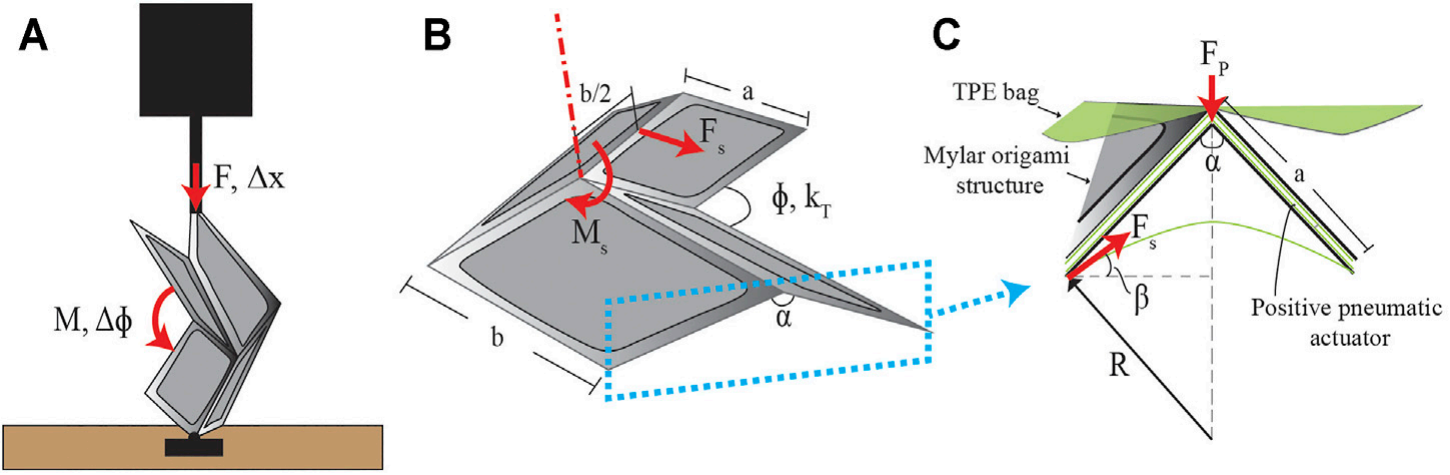
Analytical model parameters. **(A)** Experimental setup for determining the torsional spring constant, *k*_*T*_, of a single origami unit. A single origami unit is mounted to a plate on its hinge so that it can be folded by the applied displacement Δ*x*. **(B)** The parameters used to model a single Miura-Ori unit. The surface tension force, *F*_*s*_, causes the moment, *M*_*s*_, about the angle, *ϕ*. **(C)** A zoomed-in cross section view of where the force due to surface tension is modeled, along with the necessary parameters, due to the TPE film that encloses the origami pattern.

Multiple approaches have been used in the literature to model fluidic-driven soft origami structures (Li et al., 2017; Gollob et al., 2021). To analytically model the retractor, we assume that the origami sheet is wrapped in an ideal cylindrical configuration and that, by symmetry, the moment balance of a single section of the origami can be applied to the whole retractor. We first model the relationship between the vacuum pressure in the retractor and its expansion. The flexure joint of a single Miura-Ori unit was modeled as a torsional spring that provides a restoring elastic moment. Using the parameters shown in Figure 4B, a moment balance was performed showing that this restoring moment is equivalent to the moment, *M*_*s*_, that is produced due to surface tension of the TPE film encapsulating the origami pattern:

$$k_{T}\Delta \phi =M_{s}$$ (1)

where *k*_*T*_ is the torsional spring constant of an origami unit, Δ*ϕ* is the change in angle induced by *M*_*s*_. The force *F*_*s*_, that causes *M*_*s*_, is modeled using the law of LaPlace: Δ*P* = *γ*/*R*, which gives the difference in pressure, Δ*P*, as the surface tension in force per unit length, *γ*, multiplied by the inverse of the radius of curvature of the TPE covering the origami, *R* (Figure 4C). The force is obtained by solving for *γ* and multiplying by the length along which the surface tension acts. The force due to surface tension can then be found as:

$$F_{s}=\Delta PRb$$ (2)

where *b* is the long dimension of the origami unit (Figure 4B). The direction of this force is tangent to the films curvature defined by the angle, *β*, to the horizontal. This angle is defined by:

$$\beta =\cos^{-1}\frac{a\sin\frac{\alpha_{0}}{2}}{R}$$ (3)

where *a* is the short dimension of the origami unit, *α*_0_ is the initial dihedral angle, and *R* is obtained by approximating the TPE film as a parabola with points at each flexure joint and a measured film height. cos *β* projects the force onto the top plane that intersects the joints of the origami, as shown in Figure 4C. Using this to solve the moment balance in Eq. 1, the angle of a single origami unit at a given vacuum Δ*P* can be defined as:

$$\phi =\frac{\Delta PR\frac{b^{2}}{2}\cos\beta }{k_{T}}+\phi_{0}$$ (4)

where *ϕ*_0_ and *k*_*T*_ are both determined experimentally. The moment arm of *M*_*s*_ is *b*/2 (Figure 4B). Force-displacement data was obtained for a single Miura-Ori unit including all the material layers outlined in Section 2.3. The origami unit was oriented as shown in Figure 4A. The single unit was fixed at the edge of its flexure joint using a laser cut fixture under an Instron testing machine (5943 Instron, United States) equipped with a 50 N load cell (Instron 2530-50N, United States). Testing was performed using an input displacement of 5 mm at 10 mm/min speed. This data was analytically transformed into moment-angular displacement data. The Curve Fitting Toolbox in MATLAB was used to fit a nonlinear power curve to the data. *k*_*T*_ was calculated by taking the derivative:

$$k_{T}=\frac{dM}{d\phi }.$$ (5)

The spring constant was multiplied by a corrective factor of 2.5 in all calculations in order to account for the multiple flexure joints in parallel at any given location on the retractor’s circumference. For the proposed design, there are five joints in parallel; however, while each is attached to the other, each joint also experiences its own forces. Therefore, the corrective factor of 2.5 was derived as half the number of flexure joints along the axis of the cylindrical retractor (Figures 1A,B, side view). To approximate the second constant, *ϕ*_0_, the total circumference was assumed to be approximately equivalent to the sum of the lengths of each origami unit. An initial experimental measurement of the retractor diameter, *D*_0_, and the design parameters *b* (the long origami dimension) and *n* (the number of origami edges along the whole circumference), were used to calculate the initial angle, *ϕ*_0_ using:

$$\phi_{0}=2\sin^{-1}\frac{D_{0}\pi }{nb}$$ (6)

and subsequently, through geometric relations, *α*_0_, used in Eq. 3. With all parameters defined in Eq. 4, the resulting *ϕ* can be related to a diameter, *D*, using the same relation in Eq. 6 and solving for *D* to get:

$$D=\frac{nb}{\pi }\sin\frac{\phi }{2}.$$ (7)

The ratio *D*/*D*_0_ describes the expansion of the cylinder for vacuum pressures *P* < 0.

We then model the expansion of the retractor due to positive pressure. The TPE pneumatic actuators embedded in the structure are centered on a single unit in such a way that the only force produced would be in the radial direction (Figures 2C,D). Therefore, a force is produced at the flexure joint of a given unit and causes a moment on the two neighboring flexure joints. The force due to positive pressure is:

$$F_{P}=\frac{\Delta PA}{2}$$ (8)

where *A* is the area of the pneumatic actuator created by the Teflon mask (see Section 2.3). Since the pressure provides force in both directions, we only look at the force outward so we divide this by two. This moment balance is found to be:

$$2k_{T}\alpha =F_{P}a\sin\frac{\alpha_{0}}{2}$$ (9)

where *α* is used as the angle instead of *ϕ* because the positive actuator directly affects *α*, and it is assumed that *k*_*T*_ resists changes in *α* and *ϕ* uniformly since one can be translated into the other. The moment arm, *a* sin(*α*_0_/2), is the short dimension of the origami projected perpendicular to the force *F*_*P*_ (Figure 4C). The same experimentally derived *k*_*T*_ as described above was used. Eq. 9 can be solved for *α* and then translated to *ϕ* using geometric relations. This can be solved similarly to what was done for vacuum pressures by substituting *ϕ* into Eq. 7 and obtaining a ratio, *D*/*D*_0_, that gives expansion that is valid for *P* > 0. Because of the manufacturing process described in Section 2.3, the retractor is not an ideal cylinder as assumed by the model and takes on an elliptic cross-section upon expansion. Thus, we can find that this ratio describes the major axis expansion of the retractor and does not capture minor axis expansion. Combining the calculated ratios of *D*/*D*_0_ in the vacuum and positive pressure regimes gives the total expansion for the major axis of the retractor: *L*_*major*_/*L*_0_ = *D*/*D*_0_.

To model the force output of the retractor, a force balance was used for both positive and vacuum pressures. We assume that the system is static so that we can model approximate forces based on the initial folded configuration of a single origami unit in the retractor. The average radial force output will be modeled to represent the interaction between the retractor and brain tissue. The force which contracts the retractor is the same force from Eq. 2. To obtain the radial component of this force, a series of transformations into Cartesian and then, into polar coordinates, is performed. The resulting relation is:

$$F_{r}=\Delta PRb\left(\sin\beta \sin2\theta +\cos\beta \right)$$ (10)

where *θ* is the coordinate on the circumference of the cylindrical retractor. To obtain the average, *F*_*r*_ was calculated for values of *θ* ∈ [0, *π*/2] and averaged since it is assumed that the retractor is an ideal cylinder and symmetry applies. Similarly, the average elastic force due to the torsional spring was modeled by using the angular displacement values that were calculated at each pressure using Eq. 4 in:

$$F_{elastic}=\frac{k_{T}\Delta \phi }{b}\left(\sin\frac{\phi }{2}\sin2\theta +\cos\frac{\phi }{2}\right).$$ (11)

The overall radial force output for the retractor can now be modeled as:

$$F_{output}=F_{r}-F_{elastic}.$$ (12)

The positive actuation of the retractor is achieved through TPE pneumatic actuators placed between two layers of flexible Mylar. The positive pressure applied within these actuators causes greater radial forces after the retractor has expanded. This relationship is the same as Eq. 8. Similar to Eq. 12, the total output force for positive pressure would be:

$$F_{output,P}=F_{P}-F_{elastic}$$ (13)

with the same average values for *F*_*elastic*_. The final equation for the overall force output across both the negative and positive pressure regimes is:

$$F_{expand}=\left\{\begin{array}{c} F_{output}\;\Delta P\leq 0\;\\ {\left.F_{output,P}+F_{output}\right|}_{\Delta P=0}\;\Delta P>0\; \end{array}\right.$$ (14)

To obtain the contraction force of the retractor, a force balance was performed on a single unit. The force that the unit will contract with is given as:

$$F_{contract}=2F_{s}\sin\beta .$$ (15)

This equation is true for Δ*P* < 0 and can be thought of as the force with which the retractor will pull on tissue if it were anchored on the opposite end.

## 3 Experiments

### 3.1 Force Characterizations

We characterized maximum blocked force output of the retractor during expansion and contraction. Expansion force directly relates to the clinical function of the retractor to displace brain tissue and open a passage for surgical tools. Contraction force does not relate to a specific clinical function and was rather tested for mechanical performance characterization.

**FIGURE 5:**
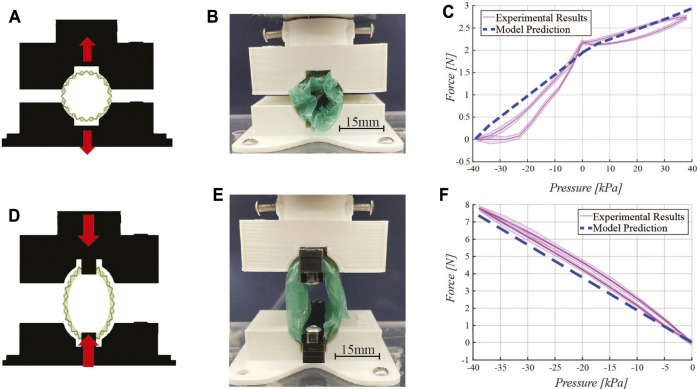
Force characterizations. Expansion force testing: **(A)** schematic diagram, **(B)** experimental setup, and **(C)** experimental results and model prediction. Contraction force testing: **(D)** schematic diagram, **(E)** experimental setup, and **(F)** experimental results with the model prediction. In both force characterizations, a 3D printed fixture constrains the retractor onto the Instron testing machine load cell as negative and positive pressure is delivered to the pneumatic chambers of the device.

This experiment was completed on an Instron testing machine (5943 Instron, United States) equipped with a 50 N load cell (Instron 2530-50N, United States). A 3D-printed fixture for the retractor was designed in PLA using an Ultimaker S3 printer (Ultimaker, Geldermalsen, Netherlands), as shown in Figures 5B,E. The prototype is placed in two distinct configurations for compression and tensile testing, respectively (Figures 5A,D). The same fixture is used for both types of testing. The retractor is supplied regulated pressure using an electro-pneumatic control system consisting of an ITV900 series vacuum regulator (SMC Corporation, Tokyo, Japan) supplied with vacuum pressure, in the case of negative pressure, and an ITV0010 series pressure regulator (SMC Corporation, Tokyo, Japan) connected to a compressed air line, for positive pressure. In both cases, pressures are monitored with an ADP5 series piezoelectric sensor (Panasonic Industry, Osaka, Japan). Data acquisition and control interface is provided using an NI 782 602–01 multi-function DAQ I/O (National Instruments, Austin TX, United States). A LabView user interface allows the control of the vacuum pressure as data is collected.

For expansion force testing, the fully-contracted retractor is placed in the fixture while a vacuum is pulled to reach its minimum diameter. The fixture is positioned such that the retractor will not move when the vacuum is released, as shown in Figure 5B. The test begins by releasing vacuum pressure in incremental steps, from a minimum of −40 kPa to atmospheric pressure, in steps of 5 kPa. The expanding device pushes against the fixture applying a force which is detected by the load cell of the Instron. At atmospheric pressure, positive pressure is directed into the pneumatic actuator pockets integrated in the origami structure. Positive pressure is tested from 0 to 40 kPa in 5 kPa steps. Return cycles from 40 kPa to −40 kPa in steps of 5 kPa were also characterized.

For contraction force testing, force is measured by fixing two ends of the retractor to the Instron fixture, as shown in Figure 5E. The test begins at atmospheric pressure. In steps of 5 kPa, force is recorded down to -40 kPa. The positive pressure actuation component is not included in this test. Return cycles from 0 kPa to −40 kPa in steps of 5 kPa were also characterized.

### 3.2 Motion Characterization

**FIGURE 6:**
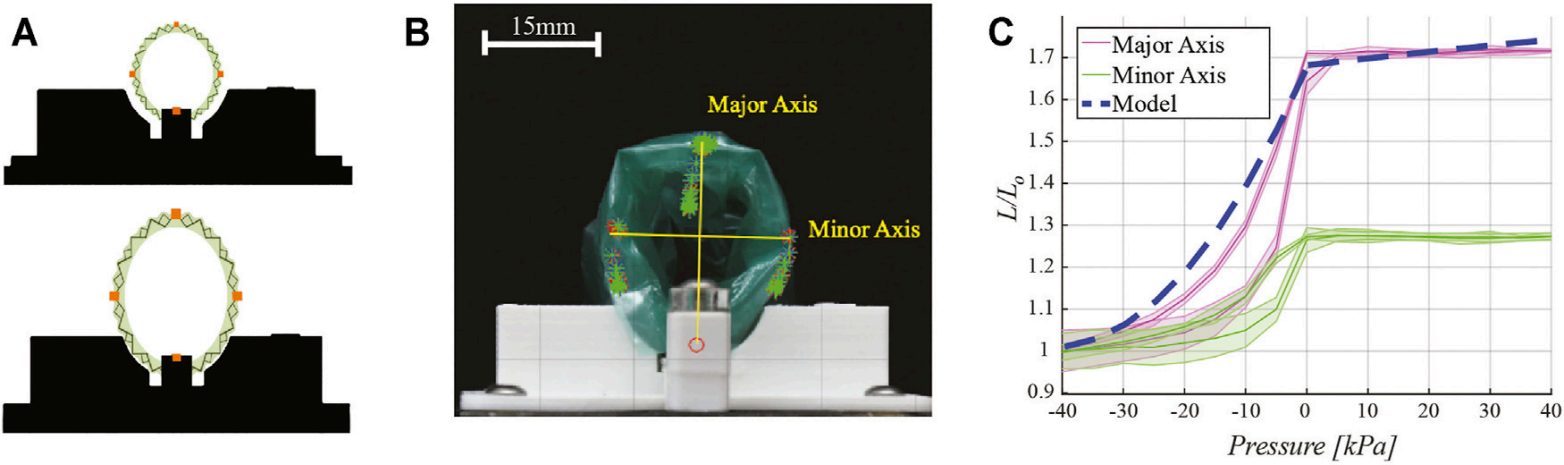
Motion characterization. **(A)** Schematic drawing of motion capture testing setup indicating the placement of retro-reflective tracking dots (in orange) placed on the retractor perimeter. **(B)** Experimental testing setup. Images captured at various pressures show the displacement of the tracking points, which form the major and minor axis of an ellipse. Overlaid yellow lines for major and minor axes are reported for clarity of presentation. **(C)** Plot of retractor motion upon delivery of pressure, showing major and minor axes expansion, normalized to the length of the axes at −40 kPa, experimental results and model prediction which was given as *D*/*D*_0_.

Retractor expansion and contraction upon pressure delivery was characterized. The experimental setup uses retro-reflective tracking points physically placed in four different locations along the perimeter of the structural origami layer of the retractor (Figure 6A). The device is fixed on one end in a 3D-printed fixture and allowed to freely move with respect to this anchoring point, as shown in Figure 6B. In steps of 5 kPa, the retractor is actuated from −40 kPa to 40 kPa using the pneumatic control circuit previously described. Pressures below −40 kPa and above 40 kPa do not cause respectively further contraction and expansion of the retractor and therefore are not of interest. These pressures are within the range of operation of other pneumatically-driven soft surgical robots (Runciman et al., 2019; Cianchetti et al., 2018; Gifari et al., 2019). Return cycles from 40 kPa to −40 kPa in steps of 5 kPa were also characterized. At each step, the system is allowed to reach steady state and a picture is taken.

These photos are then analyzed in MATLAB (MathWorks, Natick MA, United States) using a color mask filtering function, which isolates the tracking regions. The shape of the retractor was approximated as an ellipse. The centroid of each tracking region is then calculated and the length of major and minor axis of the ellipse is computed as the euclidean distance between the centroids of the markers.

### 3.3 *In-Vitro* Testing

**FIGURE 7:**
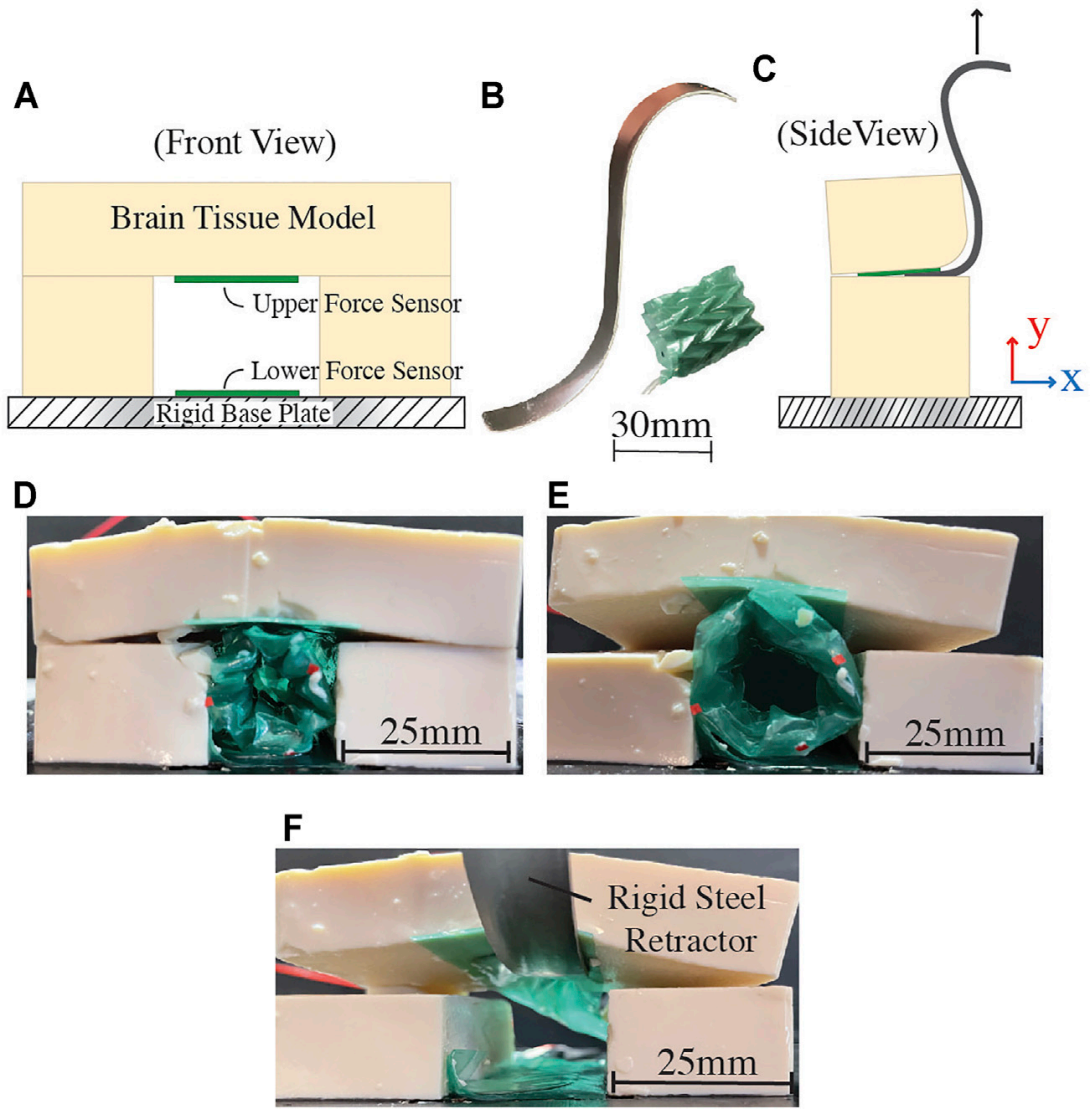
*In-vitro* testing setup and results. **(A)** Schematic of testing setup, indicating the placement of force sensitive resistors (FSRs) for data collection. **(B)** Traditional stainless steel retractor and proposed soft robotic retractor. **(C)** Test schematic with stainless steel retractor. **(D)** Soft robotic retractor inserted into the setup at −40 kPa. **(E)** Soft robotic retractor expansion at a pressure of 40 kPa. **(F)** Brain tissue model retraction using a traditional stainless steel retractor. In this case, the stainless steel retractor is only in contact with one FSR.

The soft robotic retractor is designed to gently move delicate brain tissue. Traditional stainless steel tools can induce damaging focal pressure at locations where the tool has sharp discontinuities, such as the edge of a spatula retractor. To demonstrate the potential improvements of this retractor design over traditional metallic devices, we tested our prototype on an *in-vitro* brain model (Figure 7).

The model simulates the soft, compliant properties of brain tissue using tofu (Morinaga America Inc., Irvine CA, United States). For this experimental setup, the retractor is positioned adjacent to pre-cut layers of tofu on three sides. The fourth side is the rigid base plate of the experimental setup. The system is constructed in this way to simulate a potential use-case inside the brain, where one side of the retractor uses the rigid bone of the cranium as a mechanical anchoring point (Figures 1C–E).

The setup contains two force sensitive resistors (FSR 402, Interlink Electronics Inc., Irvine CA, United States), one on the grounding surface and another at the interface between the retractor and the brain model, as shown in Figures 7A,C. These sensors gather force data as the retractor expands and displaces the simulated brain tissue. Experiments were conducted at -40, 0, and 40 kPa. For comparison, this test is also conducted with a traditional stainless steel surgical retractor, pictured in Figure 7B. The force data from both the rigid and soft robotic retractors are compared by examining how much force was placed on each sensor. In each case, the brain tissue model is displaced 5 mm in the *y* direction, as indicated in Figure 7C. These tests were setup to determine the maximum forces that the soft robotic retractor and the traditional stainless steel retractor impose on the brain tissue model.

## 4 Results and Discussion

Three distinct prototypes were constructed for each test described in this section and each of them was tested three times. In the characterization results plots of Figure 5C, Figure 5F, and Figure 6C, the solid line is the mean value of the tests and the shaded area represents one standard deviation.

### 4.1 Force Characterizations

Results for expansion force and contraction force testing are presented in Figure 5C and Figure 5F, respectively.

During the expansion force characterization, the force applied by the retractor goes from 0 to 2.18 N, upon release of vacuum pressure from −40 kPa to 0 kPa. When positive pressure is delivered to the device (in the pneumatic actuators embedded in the origami structure), the force output increases up to 24%, up to a maximum of 2.7 N. Both regions exhibit a non-linear relationship between force and pressure. The dashed line in Figure 5C represents the output from the model described in Section 2.4. The model captures the trend and estimates a similar force range as the pressure varies, with an average discrepancy of 18% between the model and the experimental data. However, the model does not fully capture the nonlinearity of the data. While the torsional spring constant is nonlinear, there are other considerations such as the constraint of the TPE film limiting the maximum amount of force and the nonlinearity of the angle *β* which was taken as a constant in our model. This angle is actually dependent on the current angular configuration of the origami and thus, changes the amount of force produced at a given pressure. In other words, there is a coupling between the force given by Eq. 10 and *β*. Our model simplifies the coupling to make prediction of the forces and expansion more straightforward. The assumption of a quasi-static system allows the model to rely on a force that only changes due to pressure. Therefore, error in the predicted forces likely arises from the approximations made in Section 2.4 that the retractor is an ideal cylinder that expands uniformly in a quasi-static manner.

Contraction force testing results (Figure 5F) show a roughly linear relationship between pressure and force output. The maximum force output is 7.8 N.

### 4.2 Motion Tracking Characterization

Motion tracking test results are presented in Figure 6C, with the shape of the retractor represented by the major and minor axis of an ellipse (Figure 6B). Lengths are normalized to the axis length (*L*_0_) when the retractor pressure is -40 kPa. Results show the major axis length increases by 70% at maximum pressure. The minor axis increases by 28%. The eccentric expansion of the device is a result of the fabrication process. The flat sheet of origami is bonded at one seam to form the cylindrical retractor (Figure 3C, step 2). The retractor does not expand further once pressures above 0 kPa are delivered to the pneumatic actuators chambers; however, this confers the device a 24% larger force output, as shown in Figure 5C.

The dashed line in Figure 6C represents the output from the model described in Section 2.4. The model follows a similar trend as the data for the major axis, with a 9% maximum discrepancy. Since the model assumes the retractor expands as a perfect cylinder, this behavior is expected because uniform expansion would more closely follow the expansion of the major axis. The deviation at lower pressures is likely due to the thickness of the TPE film and the other materials used for the construction of the device, which prevent the origami from folding further at higher vacuum pressures. Upon increasing pressure towards 0 kPa, the model captures the experimental data more closely. In the range of 0–40 kPa, the model predicts a slight increase in the retractor diameter; however, this increase is not observed in the experimental data. One likely reason is because of the direct adhesion of the surface of the actuators to the flexure Mylar layers during fabrication. This would result in no overall moment since both sides of the actuator are fixed to move with the origami pattern. There is also a constraining layer of TPE around the whole retractor which limits the amount of expansion past atmospheric pressure.

### 4.3 *In-Vitro* Testing and Comparison with Traditional Stainless Steel Retractor

Force characterization done in the *in-vitro* testing setup shows the soft robotic retractor’s reduced force output on the brain tissue model as compared to the traditional stainless steel retractor used in the same experimental setup. The testing setup is designed to replicate the use-case when the soft retractor is braced against the rigid surface of the cranial bone, as seen in Figures 1C–E, and uses this surface as an anchoring point for the retractor to push into soft neurological tissue. The most clinically significant interactions in this use-case are with the sensitive neurological tissue rather than cranial bone. This is because, as discussed in Section 1, traditional retractors can generate excessive pressure on brain tissue during surgery and consequent tissue damage, leading to neurological injuries and other functional impairments post-surgery (including aneurysms, brain edema, vascular compromise causing ischemia, and direct damage to the surrounding cortex) (Zhong et al., 2003). This happens both in skull base and intracranial surgical operations (Andrews and Bringas, 1993). Furthermore, traditional brain retractors are typically being mounted or anchored to the skull during neurosurgical procedures through drilled burr-holes (Zagzoog and Reddy, 2020).

Test results are presented in Table 1. The proposed retractor produced a maximum force of 0.74 N on the lower sensor, which is braced against the rigid base plate of the test setup simulating the cranial bone, as shown in Figures 7D,E. This force is less than the maximum force reported for typical tool-bone interactions of up to 0.82 N during skull base procedures (Bekeny et al., 2013). The maximum force produced on the brain tissue model was 0.2 N. Figure 7F shows the traditional stainless steel retractor used in the same setup. In this case, the traditional stainless steel rigid retractor produced a maximum force of 0.96 N on the brain tissue model. The proposed soft device shows a reduction of 4.8 times of the force applied onto the brain tissue model with respect to the traditional rigid retractor to generate the same displacement. The forces produced on the rigid base plate are not of clinical significance as this surface represents a rigid anchoring point on the cranial bone, as illustrated in Figure 1. In this study, we are interested in reducing the interaction forces between the surgical tool and delicate brain tissue. At each tested actuation pressure, the retractor produced forces on both surfaces that were lower than the single force produced by the stainless steel retractor.

**TABLE 1:** *In-Vitro* comparison between rigid stainless steel retractor and soft robotic retractor.

	Pressure [kPa]	Upper sensor force [N]	Lower sensor force [N]
Soft Retractor	−40	0.032	0.032
Soft Retractor	0	0.144	0.199
Soft Retractor	40	0.206	0.740
Stainless Steel Retractor	N/A	0.963	0.000

## 5 Conclusion

In this paper we reported on the design, fabrication, modeling, and characterization of a novel soft robotic retraction tool for neurosurgical applications. The design of an expandable origami structure, actuated through the delivery of positive or negative air pressures, is presented. Expandable origami structures are used to leverage their small scale and deployable function in neurosurgery where space is a limiting factor. Such a device may be used to gently open a surgical corridor, allowing visualization of brain tissue and cerebrovascular structures and working room for their safe manipulation. Surgical workspace generation can be tuned through the controlled application or release of pressures, allowing for granular control of actuation in the brain.

The soft robotic retractor measures 25.2 mm in outer diameter when fully expanded and 14.8 mm when fully contracted with vacuum pressure. These dimensions are appropriate for the proposed surgical application. The manipulation of narrow corridors with microinstrumentation under a microscope are the mainstay of work in cranial tumor surgery and skull base surgery, where avenues may be as small as 1 cm and instruments are used within this space. The proposed device can be used also with intracranial endoscopy instrumentation (which is part of routine neurosurgical procedures), as the outer diameter of commercially available scopes is typically less than 5 mm (Ho and Hwang, 2015). Further, the use of such a device may minimize the need for surgical assistants to manually apply traction. The surgeon may insert the retractor with the device needing minimal attention while in operation. Having a controllable robotic device to assist during cranial surgery can potentially mitigate risks associated with traditional rigid metallic retractors. The proposed system can perform similarly to commercially available rigid retractors (currently in clinical use) in terms of tissue displacement capability. Therefore, it has the potential to be easily integrated in the clinical/surgical workflow without disrupting or drastically changing current surgical methodologies and techniques. The main key difference and unique strength of the proposed device rely on the fact that it is soft as well as that it can be positioned and then controlled to be deployed/inflated; as opposed to traditional retractors, which are rigid and fixed and therefore more likely to be traumatic. At the conclusion of the procedure, the surgeon can depressurize the device for its safe removal. Characterizations completed on the retractor show the capability of the device to move soft tissue, and *in-vitro* testing demonstrates the benefits of the soft device in lowering forces applied to a brain tissue model over traditional, rigid stainless steel surgical retractors.

The soft robotic retractor operates at relatively low pressures of actuation (ranging from −40 kPa to 40 kPa), which is within the pressure ranges from other soft surgical robots (Runciman et al., 2019). This work presents a relatively low-cost, easy to manufacture device that can be customized to patient anatomy, upon consultation with neurosurgeons and skull base surgeons in the pre-operative and planning phase. Soft pneumatic actuation is considered clinically safe for the neurosurgical procedures targeted by the robotic retractor. This is because surgeons operate via an open cavity in the cranium which is exposed to air at atmospheric pressure allowing for a vent in the case of a leak. Furthermore, pneumocephalus (i.e., the presence of air in the intracranial space, which can occur following trauma, cranial surgeries, or spontaneously) after cranial surgery is not harmful, and self-resolves or can be treated with minimal maneuvers like high flow oxygen (Cunqueiro and Scheinfeld, 2018; Siegel et al., 2018).

Future work will target *ex-vivo* characterizations of this device with animal models and will also focus on the integration of soft sensing elements for real-time pressure and physiological condition feedback. Further modification of the design and fabrication process will be conducted so that our model can better represent the behavior of the retractor and inform its control. We will explore the use of *C O*_2_ gas to inflate the retractor for added safety precautions. Also, we will explore further miniaturization of the device for trans-nasal skull base surgical applications.

## Data Availability

The raw data supporting the conclusions of this article will be made available by the authors, without undue reservation.
